# Association between thyroid autoimmunity and gestational diabetes mellitus in euthyroid women

**DOI:** 10.1530/ETJ-21-0142

**Published:** 2022-02-22

**Authors:** Georgiana Sitoris, Flora Veltri, Malika Ichiche, Pierre Kleynen, Jean-Philippe Praet, Serge Rozenberg, Kris G Poppe

**Affiliations:** 1 Endocrine Unit Centre Hospitalier Universitaire Saint-Pierre, Université Libre de Bruxelles (ULB), Brussels, Belgium; 2 Department of Internal Medicine, Centre Hospitalier Universitaire Saint-Pierre, Université Libre de Bruxelles (ULB), Brussels, Belgium; 3 Department of Gynecology and Obstetrics, Centre Hospitalier Universitaire Saint-Pierre, Université Libre de Bruxelles (ULB), Brussels, Belgium

**Keywords:** gestational diabetes, pregnancy, thyroid autoimmunity, euthyroid

## Abstract

**Objective:**

Pregnant women with autoimmune (subclinical) hypothyroidism have an increased risk of developing gestational diabetes mellitus (GDM). However, this association remains controversial in euthyroid women with thyroid autoimmunity (TAI). Therefore, the aim of the study was to determine the association between TAI and GDM in euthyroid women in a logistic regression analysis with adjustments for baseline/demographic parameters.

**Methods:**

Cross-sectional study in 1447 euthyroid women who performed their entire clinical/biological workup and oral glucose tolerance test (OGTT) in our center. At median 13 (11–17) weeks of gestation, thyroid-stimulating hormone, free T4, and thyroid peroxidase antibodies (TPOAb) were measured, baseline characteristics were recorded, and an OGTT was performed between 24 and 28 weeks of pregnancy. Exclusion criteria were pre-pregnancy diabetes, assisted pregnancies, and women with (treated) thyroid dysfunction before or after screening. The diagnosis of GDM was based on 2013 World Health Organization criteria, and TAI was defined as TPOAb levels ≥60 kIU/L.

**Results:**

Two hundred eighty women were diagnosed with GDM (19.4%), 26.1% in women with TAI, and 18.9% in women without TAI (*P*  = 0.096). In the logistic regression analysis, TAI was associated with GDM in women older than 30 years (adjusted odds ratio 1.68 (95% CI, 1.01–2.78); *P*  = 0.048). Maternal age >30 years, pre-pregnancy BMI ≥30 kg/m^2^, and other than Caucasian background were also associated with GDM; aOR 1.93 (95% CI, 1.46–2.56); *P*  < 0.001, 2.03 (95% CI, 1.46–2.81); *P*  < 0.001 and 1.46 (95% CI, 1.03–2.06); *P*  = 0.034, respectively.

**Conclusions:**

In older pregnant women, the presence of TAI in euthyroid women was associated with GDM. In line with the literature data, (higher) age and BMI were strongly associated with GDM. Future investigations should focus on treatments that might prevent the development of GDM in euthyroid women with TAI.

## Introduction

Thyroid autoimmunity (TAI) and (subclinical) hypothyroidism (SCH) have been associated with adverse pregnancy outcomes, such as miscarriage, preterm birth, and gestational diabetes mellitus (GDM) (1). TAI and GDM have been linked with each other via two pathways. One is by the development of (sub)clinical hypothyroidism (TAI is the most frequent cause of hypothyroidism) and another via inflammatory pathways involving IL-6 and TNF-α; both pathways can lead to insulin resistance (IR) (2, 3).

In a meta-analysis pooling studies from the period, 2000 to 2014, no increased risk of GDM was reported in euthyroid women with TAI (relative risk (RR) 1.07 (95% CI, 0.96–1.19)) (2). However, in original studies published since 2015 in euthyroid Chinese populations, a significant association between TAI and GDM was observed (odds ratios (ORs) between 1.65 and 2.54) (4, 5, 6). Also in the most recent meta-analysis, increased thyroid peroxidase antibodies (TPOAb) were associated with GDM (OR 1.65 (95% CI, 1.13-2.40); *P*  < 0.001). However, it should be noted that the index of heterogeneity (I^2^) was high with 74% (7). The reason for that heterogeneity was not specified and might be due to different definitions of thyroid dysfunction, changes in the criteria for the diagnosis of GDM (before and after the criteria of The International Association of Diabetes and Pregnancy Study Groups), the inclusion of different populations (cosmopolitan vs regional), and different exclusion criteria. Furthermore, in most studies, it is not clear to what extent the impact of TAI on GDM was adjusted for other variables such as (over)weight and (high) age (8).

Therefore, the aim of this study was to investigate in an adjusted logistic regression the association between TAI and GDM in euthyroid women that performed their biological workup and obstetric follow-up in a single center throughout the entire pregnancy.

## Materials and methods

### Overall study design/definitions

The obstetric clinic of the CHU Saint-Pierre is part of a downtown public university hospital in Brussels, Belgium. In this cross-sectional analysis (period February 1, 2013/December 12, 2014), we included women with ongoing pregnancies, that performed their biological work-up and obstetric follow-up in our center throughout the entire pregnancy. The oral glucose tolerance test (OGTT) is done systematically in all pregnant women in our center, and therefore also in all women included in this study.

Exclusion criteria were women with known diabetes mellitus before pregnancy, pregnancies resulting from assisted reproduction, multiple pregnancies, and (treated) thyroid disorders (with LT4/antithyroid drugs) before or after screening. These exclusions were based on previous studies on the impact of thyroid disorders and other variables on glucose metabolism as reviewed by Biondi *et al.*in 2019 (3).

**Figure 1:**
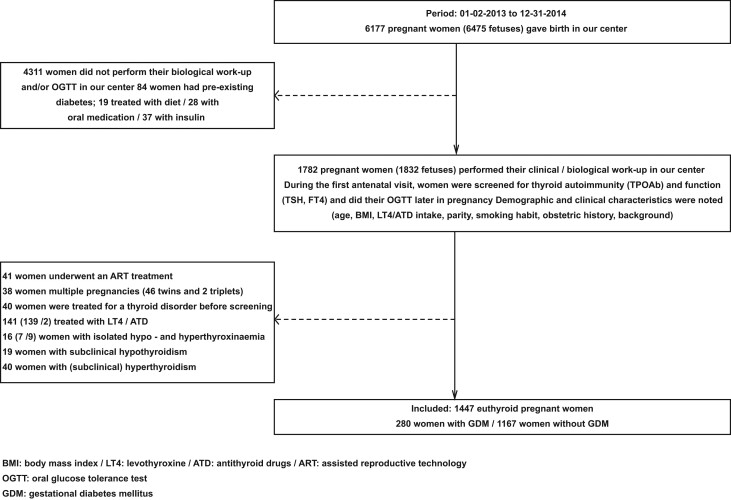
Flowchart of the study selection process.

Finally, 1447 women were included to investigate the association between TAI and GDM in euthyroid women. In Fig. 1, we illustrate in a flowchart the study selection process (noteworthy is that among all excluded women, 16.7% were TAI+ (56/335)).

During the first antenatal consultation, demographic and obstetrical data are noted and systematically completed with a biological analysis including serum thyroid-stimulating hormone (TSH), free thyroxine (FT4), TPOAb, and ferritin measurement. The ethnic background of the women is based on a history taken by the social workers that includes systematically the nationality at birth and the ethnic origin of the women (9). Gestational age is based on ultrasound findings and expressed in full weeks and days of amenorrhea. Smoking is stratified as yes/no (yes meant a minimum of five cigarettes daily and women who stopped smoking during pregnancy were also considered as smokers).

TAI is present when TPOAb levels were ≥60 kIU/L. In a previous study, we determined the first-trimester reference range (2.5–97.5th percentile) for serum TSH (0.06–3.74 mIU/L) and FT4 (10.29–18.02 pmol/L) (10). GDM is diagnosed after the administration of 75 g glucose during an OGTT performed between 24 and 28 weeks of pregnancy when fasting glucose ≥92 mg/dL or 1-h postprandial glycemia ≥180 mg/dL or 2 h ≥153 mg/dL) (11, 12). Iron deficiency is defined as serum ferritin levels <15 µg/L.

The study was approved by the institutional review board (AK/15-11-114/4568); no written consent was obtained from the participants.

### Serum assay

All provisions were implemented by the laboratory of hormonology of our institution.

Serum TSH, FT4, TPOAbs, and ferritin levels were measured using the Chemiluminescence Centaur XP Siemens immunoanalyzer. The reference values were 0.3–4.0 mIU/L, 10.3–25.7 pmol/L (0.8–2.0 ng/dL), <60 kIU/L, and 15–300 ug/L for TSH, FT4, TPOAb, and ferritin, respectively. The total imprecision CVs were 6.9, 4.2, 7.6, and 3.7% for TSH, FT4, TPOAb, and for ferritin, respectively. Plasma glucose was measured by an automated colorimetric-enzymatic method on a Hitachi/Roche-Modular P analyzer; CV is 1%.

### Statistical analysis

Data were stored in a Microsoft Excel database and statistical analyses were performed using Stata 11.2 software (StataCorp LLC, College Station, TX, USA). Differences between groups were analyzed by Fisher’s exact tests for categorical data and by a *t*-test or Mann–Whitney *U* test for continuous data. A multivariable logistic regression analysis was performed with GDM as a dependent outcome. As independent outcomes, we included variables with a proven impact on GDM (6, 8). These are: maternal age (whole range and as higher age (>30 years)), pre-pregnancy BMI (whole range and as obesity (≥30 kg/m^2^)), background other than Caucasian (e.g. Sub-Saharan and North African), tobacco use, ferritin levels (whole range and as iron deficiency (ferritin levels (<15 µg/L)), parity (whole range and as high parity rate (>2)), the fetal gender (female) and TPOAb (whole range and as TAI (TPOAb ≥60 kIU/L)). The regression analysis was done ones with the independent variables as continuous data and ones with the independent variables as categorical data (the results are shown in one table). Results are expressed as adjusted odds ratios (95% CI), and statistical tests were considered significant whenever *P*  < 0.05.

## Results

Table 1 shows the demographic and obstetric parameters in all women and according to the presence (GDM+)/absence (GDM−) of gestational diabetes.

**Table 1:** Demographic and obstetric parameters in all women and according to the presence (GDM+)/absence (GDM−) of gestational diabetes.

Continuous data^a^	All women	GDM+	GDM−	*P*
Categorical data, *n* (%)	*n*= 1447	*n*= 280 (19%)	*n*= 1167 (81%)	
Maternal age (years)	30.0 ± 5.8	31.7 ± 5.4	29.5 ± 5.8	<0.001
Maternal age >30 years	665 (46.0%)	170 (60.7%)	495 (42.4%)	<0.001
Pre-pregnancy BMI (kg/m^2^)	25.0 (22.3–28.2)	26.8 (23.3–30.0)	24.6 (22.1–27.6)	<0.001
Obesity (BMI ≥30 kg/m^2^)	228 (15.8%)	71 (25.4%)	157 (13.5%)	<0.001
Other than Caucasian background	1103 (76.2%)	230 (82.1%)	873 (74.8%)	**0.010**
Parity (*n*)	1 (0–2)	1 (0–2)	1 (0–2)	**0.007**
Multiparity (>2)	177 (12.2%)	43 (15.4%)	134 (11.5%)	0.076
History of ≥2 first-trimester MC	96 (6.6%)	22 (7.9%)	74 (6.3%)	0.360
Smoking during pregnancy	218 (15.1%)	36 (12.9%)	182 (15.6%)	0.250
Ferritin levels (μg/L)	20 (12–37)	23 (13–40)	20 (12–36)	0.122
Iron deficiency (Ferritin <15 μg/L)	503 (34.8%)	87 (31.1%)	416 (35.7%)	0.149
Fetal gender (% female)	738 (51.0%)	147 (52.5%)	591 (50.6%)	0.577

^a^Continuous data are expressed as mean ± s.d. or median (Q1–Q3).

GDM, gestational diabetes mellitus; MC, miscarriage.

Bold indicates statistical significance.

Of the 1447 pregnant women included, 280 (19%) had GDM and 1167 (81%) did not.

Women with GDM were older (mean maternal age 31.7 ± 5.4 years vs 29.5 ± 5.8 years; *P*  < 0.001), more often obese before pregnancy (25.4% vs 13.5%; *P*  < 0.001) and had more often another than Caucasian background (82.1% vs 74.8%; *P*  = 0.010). Parity was higher in the GDM group, median (interquartile range (IQR)): 1 (0–2) vs 1 (0–2); *P*  = 0.007. However, when expressed as multiparty rate >2, no difference was present; 15.4% vs 11.5%; *P*  = 0.076.

Table 2 shows thyroid parameters in all women and according to the presence (GDM+)/absence (GDM−) of gestational diabetes.

**Table 2:** Thyroid parameters in all women and according to the presence (GDM+)/absence (GDM−) of gestational diabetes.

Continuous data^a^	All women	GDM+	GDM−	*P*
Categorical data, *n* (%)	*n* = 1447	*n* = 280 (19%)	*n*= 1167 (81%)	
TSH (mIU/L)	1.37 (0.88–1.89)	1.40 (0.93–1.91)	1.36 (0.88–1.89)	0.706
TSH >2.50 mIU/L	140 (9.7%)	25 (8.9%)	115 (9.9%)	0.638
FT4 (pmol/L)	14.2 (12.9–15.4)	13.5 (12.9–14.2)	14.2 (12.9–15.4)	0.441
TPOAb (kIU/L)	28 (28–37)	29 (28–38)	28 (28–37)	0.337
TAI (TPOAb ≥60 kIU/L)	88 (6.1%)	23 (8.2%)	65 (5.6%)	0.096^a^

^a^Continuous data are expressed as median (Q1–Q3).

FT4, free thyroxine; TAI, thyroid autoimmunity; TSH, thyrotropin; TPOAb, thyroid peroxidase autoantibodies.

Serum TSH levels (median (IQR)) were comparable between both study groups: 1.40 (0.93–1.91) mIU/L vs 1.36 (0.88–1.89) mIU/L; *P*  = 0.706. The prevalence of high-normal serum TSH levels (>2.50 mIU/L) was comparable between both groups: 8.9% vs 9.9%; *P*  = 0.638. Serum FT4 levels (median (IQR)) were comparable between both groups: 13.5 (12.9–14.2) pmol/L vs 14.2 (12.9–15.4) pmol/L; *P*  = 0.441. The prevalence of TAI was 8.2% in the GDM+ group and 5.6% in the GDM− group; *P*  = 0.096.

Table 3 shows the multivariable logistic regression analysis with demographic, obstetric/pregnancy parameters, and thyroid parameters as independent variables, and GDM as a dependent outcome.

**Table 3:** Logistic regression analysis with demographic, obstetric/pregnancy parameters as independent variables, and gestational diabetes as a dependent outcome.

Independent variables^a^	Dependent Outcome (GDM)	
	aOR (95% CI)	*P*
Age (whole range)	**1.06 (1.04–1.09)**	<0.001
Age >30 years	**1.93 (1.46–2.56)**	<0.001
BMI (whole range)	**1.07 (1.04–1.09)**	<0.001
BMI ≥30 kg/m^2^	**2.03 (1.46–2.81)**	<0.001
Background (other than Caucasian)	**1.46 (1.03–2.06)**	**0.034**
Tobacco use	0.90 (0.60–1.34)	0.593
Ferritin levels	1.00 (0.99–1.01)	0.328
Iron deficiency (<15 µg/L)	0.81 (0.60–1.07)	0.140
Parity (whole range)	0.95 (0.84–1.08)	0.437
High parity (>2)	1.26 (0.94–1.68)	0.122
Fetal gender (female)	1.13 (0.86–1.47)	0.384
TSH (all normal) range	1.07 (0.89–1.28)	0.497
TSH >2.50 mIU/L	1.00 (0.62–1.60)	0.990
FT4 (all normal range)	1.00 (0.92–1.09)	0.959
TPOAb whole range	1.00 (0.99–1.00)	0.811
TAI^b^	1.58 (0.94–2.66)	0.084
TAI^c^	**1.68 (1.01–2.78)**	**0.048**

^a^Given as continuous or categorical values; ^b^Age and BMI included as continuous variables; ^c^Age and BMI included as categorical variables older age and obesity.

aOR, adjusted odds ratio; GDM, gestational diabetes mellitus; POAb, thyroid peroxidase antibodies; TAI, thyroid autoimmunity (TPOAb ≥60 kIU/L).

Bold indicates statistical significance.

Significantly associated with GDM were age in the whole range, aOR 1.06 (95% CI, 1.04-1.09); *P*  < 0.001 and as higher age (>30 years), aOR 1.93 (95% CI, 1.46–2.56); *P*  < 0.001, pre-pregnancy BMI in the whole range, aOR (95% CI, 1.04–1.09); *P*  < 0.001 and as obesity (BMI ≥30 kg/m^2^), aOR 2.03 (95% CI, 1.46–2.81); *P*  < 0.001, and another other than a Caucasian background, 1.46 (95% CI, 1.03–2.06); *P*  = 0.034.

In the model with age and BMI as continuous variables, TAI was not associated with GDM, aOR 1.58 (95% CI, 0.94-2.66); *P*  = 0.084. In the model with age and BMI as categorical variables (>30 years and BMI ≥30 kg/m2), TAI was associated with GDM, aOR 1.68 (95% CI, 1.01-2.78); *P*  = 0.048.

## Discussion

The main observation in our study is the significant association between increased TPOAb levels in early pregnancy and the occurrence of GDM later on during pregnancy in women older than 30 years. Our results are in line with recently published studies performed in Chinese populations and a meta-analysis in euthyroid women with TAI in which the OR was 1.65 (95% CI, 1.13–2.40) (4, 5, 6, 7). Noteworthy concerning that recent meta-analysis is the high index of heterogeneity (I^2^of 70%), which was not specified more in detail (7). Furthermore, in many studies included, it was not clear to what extent adjustments for confounders such as age, BMI, ethnic background, subclinical thyroid dysfunction were made. Therefore, we adjusted our results for these confounders, selected patients to avoid treatments with an impact on thyroid function that were started before or after the screening, defined euthyroidism according to our institutional cut-off, and finally, included pregnant women of our cosmopolitan area. After adjustments, the association between TAI in early pregnancy and GDM persisted, but only in older pregnant women.

In a US study, it was reported that the prevalence of GDM was the highest among Filipina (10.9 %) and Asian women (10.2%), and the lowest among Caucasian (4.5%) and African American women (4.4%) (13). In our study, background other than Caucasian was associated with a higher prevalence of GDM. However, since these women had mainly a North African and sub-Saharan background (<1% far Asian), we could not specify which one was associated with a higher prevalence of GDM (10). The reasons why the prevalence of GDM differs between women according to their background are multi-faceted, including another lifestyle (physical activity/alimentary), BMI, genes associated with IR, and healthcare systems access.

A difference between our study and recent Chinese studies is the pre-pregnancy BMI that was 20–21 kg/m^2^ in theirs compared with 25.5 kg/m^2^ in ours. This might explain the higher prevalence of GDM in general in our cohort (~19%) compared with ~14% in mainland China (14). However, and despite the difference in the baseline prevalence of GDM, positivity for TPOAb remained significantly increased with GDM, independent of BMI. Obesity is a variable known to be associated with IR and GDM, as it was also the case in our regression analysis (8, 15, 16). In one study, obesity, high LDL and hyperuricemia were positively correlated with TAI in euthyroid subjects, and the so-called ‘immunometabolism’ highlights the relationship between the immune and hormonal system (17). Obesity might increase the susceptibility to harbor TAI with leptin as a peripheral determinant, which can decrease the function of regulatory T cells and increase the percentage of T helper 1 cells (18, 19). However, in a multivariable analysis in pregnant women, BMI was a poor predictor for the development of TAI (20). In line with those results, also in our study, obesity was no predictor of TAI (data not shown).

Mean age in our cohort was somewhat higher (30 years) compared with that in recent Chinese studies in which it was 28 and 27 years, respectively (5, 6). Age is a well-known variable associated with an increased risk of GDM, which we confirm in our study (4, 5, 6). When age was included as a continuous variable in the multivariable analysis, TAI was not associated anymore. Due to our strict selection criteria, 335 women were excluded of whom 16.7% were TAI+, and a type II error cannot be excluded; a priori, TAI is less frequent in younger women. Despite those exclusions, TAI remained an independent variable associated with GDM in older pregnant women. Moreover, age >30 was no predictor of TAI as such (data not shown).

The prevalence of TAI in the recent Chinese studies was higher compared with that in our cohort were 15, 9.7, and 12%, respectively, while in our study it was 6.1%; the latter maybe lower due to our strict exclusion criteria and/or the heterogeneity in the backgrounds of the women included in our cohort (4, 5, 6, 10, 21). Studies in the US and the Netherlands also showed differences in the prevalence of TAI according to different backgrounds, with the highest in white women (22, 23).

In our cohort, the prevalence of GDM increased from 18.9 to 26.1% in women with TAI, and several mechanisms have been suggested that could link TAI with GDM through IR, a key element in GDM (3, 24). One pathway is via higher serum TSH levels, since TAI is the most frequent cause of hypothyroidism, and the latter has been associated with IR as reviewed in detail by Biondi *et al.*(3). Already in infants, higher serum TSH levels have been associated with IR through a reduction in blood flow in the skeletal muscle and adipose tissue and decreased glucose uptake via lesser expression of the GLUT-4 glucose transporter (25). In a recent study, another hypothesis was brought forward to explain the association between thyroid disorders and GDM. The authors observed that lower serum human chorionic gonadotrophin (hCG) levels during the first trimester were associated with a higher prevalence of GDM, with FT4 as a mediator (26). However, in the sensitivity analysis restricted to TPOAb-positive women, hCG was not associated with GDM. In our study, we included only euthyroid women, and furthermore, the impact of TAI on GDM was adjusted for high-normal serum TSH (>2.5 mIU/L) indicating other mechanisms beyond that of a (subtle) thyroid dysfunction must be involved.

Another pathway that can link TAI and GDM is via inflammatory pathways that are common in both conditions (24, 25, 27). In one study, levels of serum interleukin‐6 (IL‐6), tumor necrosis factor‐α, IL‐12, IL‐10, and IR (HOMA‐index) were investigated in women with Hashimoto’s disease, in euthyroid women with TAI and a control group (27). These inflammatory parameters were the highest in the Hashimoto’s group but were also higher in euthyroid patients with TAI compared with those in the controls. In women with GDM, some of these parameters (IL-6, TNF-α) were also involved in the pathogenesis, and therefore, a link with TAI is plausible (24).

Finally, is it noteworthy, that in another recent meta-analysis on the association between thyroid disorders and GDM, the presence of TAI together with SCH led to the highest OR; 2.04 (95% CI, 1.32–3.13) compared to the presence of each risk factor separately (28).

An altered iron metabolism can also play a role in an association between TAI and GDM. In a previous study, we and others showed that women with low ferritin levels had a higher prevalence of TAI and mean serum TSH levels (29, 30). However, in the current analysis, ferritin levels and iron deficiency were not associated with GDM. This can be explained by the fact that high ferritin levels have been associated with GDM too, since it may serve as a marker of inflammation (associated with IR) (31, 32).

Limitations of our study are the absence TgAb levels and the lack of longitudinal measurement of thyroid function tests. In a study in the Brussels region, published a few years ago, it was shown that in infertile women, 5% had positivity for TgAb without TPOAb, and in another study in pregnant women in the same region, it was 5.9% (33, 34). Therefore, if we would have been able to include TgAb positive women, the group with TAI might have become more important and the association stronger. Indeed, in the meta-analysis discussed earlier, it was shown that the presence of increased TgAb levels only was associated with a higher prevalence of GDM compared with that in case of positivity for TPOAb; OR 1.88 (95% CI, 1.13–3.12) vs OR 1.65 (95% CI, 1.13–2.40) (28).

Concerning women with subclinical hyperthyroidism (à priori due to the high hCG levels), they might have normalized their TSH levels later in pregnancy, which cannot be confirmed due to the absence of repeated measures, and therefore, they were excluded from the study. Also, concerning euthyroid women at the beginning of pregnancy (the screening period), data in the literature suggest that they might develop SCH later during the second and third trimester (the period when the OGTT is done). In one study by Glinoer *et al.*, involving 87 euthyroid (TSH ≤4mU/L) women, TPOAb or TgAb positive, it was shown that 20% developed a serum TSH >4.0mU/L during pregnancy (35). In 2006, Negro *et al.* reported that in TPOAb-positive euthyroid women, TSH levels increased as gestation progressed, with 19% of women having supranormal (>4.0 mU/L) TSH values at delivery (36). In our series, we only had 52 repeated measures at median (Q1–Q3) 25 (23–27) weeks out of the 88 TPO+ women (first measurement 12 (11–15). At the second measurement, no woman had a TSH >3.74 mIU/L (data not shown).

Finally, the familial history of diabetes and previous episodes of GDM were not always documented in our files since some women had their GDM followed up by an endocrinologist in another center.

Strengths of the study were the exclusion of women treated with thyroid hormone or antithyroid drugs before and after the screening for thyroid disorders, and as such, avoiding their impact on the results of the OGTT performed weeks after the initial screening (at 24–28 weeks). Indeed, it has been shown in the past and in a recent study that women with TSH levels in the range 2.5–4.0 mIU/L and treated with thyroid hormones had increased rates of GDM compared with untreated women (37, 38, 39).

## Conclusion

In our cohort, applying strict inclusion and exclusion criteria, TAI in euthyroid women was associated with gestational diabetes in women older than 30 years. Furthermore, we observed a strong association with (higher) age and pre-pregnancy BMI, in line with literature data. Further research on this association is necessary, to predict better which patients with TAI in early pregnancy will develop gestational diabetes, ultimately aiming to treat them and prevent the development of gestational diabetes.

## Declaration of interest

Kris G Poppe had no conflict of interest in relation to the current study but received in the period 2018–2021 lecture fees from the Berlin-Chemie, Merck and IBSA company. The other authors have nothing to disclose.

## Funding

This work did not receive any specific grant from any funding agency in the public, commercial or not-for-profit sector.

## Author contribution statement

G S collected data and revized the manuscript; F V collected data and revized the manuscript; P K revized the manuscript; M I revized the manuscript; J P P revized the manuscript; S R revized the manuscript and approved the final version; K G P designed and performed the study, acquired and analyzed the data, drafted and revized the manuscript, and approved the final version.
